# A screen for proteins that interact with PAX6: C-terminal mutations disrupt interaction with HOMER3, DNCL1 and TRIM11

**DOI:** 10.1186/1471-2156-6-43

**Published:** 2005-08-12

**Authors:** Simon T Cooper, Isabel M Hanson

**Affiliations:** 1 University of Edinburgh, School of Molecular and Clinical Medicine, Medical Sciences (Medical Genetics), Molecular Medicine Centre, Western General Hospital, Crewe Road Edinburgh EH4 2XU

## Abstract

**Background:**

The PAX6 protein is a transcriptional regulator with a key role in ocular and neurological development. Individuals with heterozygous loss-of-function mutations in the *PAX6 *gene have malformations of the eye and brain. Little is known about the interactions of PAX6 with other proteins, so we carried out a systematic screen for proteins that interact with PAX6.

**Results:**

We used bioinformatics techniques to characterise a highly conserved peptide at the C-terminus of the PAX6 protein. Yeast two-hybrid library screens were then carried out to identify brain-expressed proteins that interact with the C-terminal peptide and with the entire PAX6 proline-serine-threonine-rich domain. Three novel PAX6-interacting proteins were identified: the post-synaptic density (PSD) protein HOMER3, the dynein subunit DNCL1, and the tripartite motif protein TRIM11. Three C-terminal *PAX6 *mutations, previously identified in patients with eye malformations, all reduced or abolished the interactions.

**Conclusion:**

Our preliminary data suggest that PAX6 interacts with HOMER3, DNCL1 and TRIM11. We propose that the interaction of PAX6 with HOMER3 and DNCL1 is a mechanism by which synaptic activation could lead to changes in neuronal transcriptional activity, and that some of the neural anomalies in patients with *PAX6 *mutations could be explained by impaired protein-protein interactions.

## Background

The PAX6 protein is a member of the PAX (paired-box) family of transcriptional regulators and is essential for normal ocular and neural development [1]. Heterozygous mutations of the human *PAX6 *gene cause aniridia (absence of the iris) and a range of other congenital eye malformations [2]. Neural defects such as foveal hypoplasia and optic nerve hypoplasia are common in *PAX6*-associated eye disease [3-5]. Homozygous mutations in man and mouse are lethal and result in severe developmental abnormalities including anophthalmia, severe reduction of the olfactory structures and gross brain malformations [2,6]. The roles of PAX6 in brain development have mainly been studied in homozygous mutant mice or rats and include arealisation of the cerebral cortex [7], formation of the prosencephalon-mesencephalon boundary [8], axon guidance [8], differentiation of neurons from glia [9] and neuronal migration in the cerebellum [10].

The discovery of multiple and diverse roles for PAX6 in brain development prompted MRI analyses of aniridia patients, and a range of distinctive brain anomalies were uncovered. The most common and striking of these was absence or hypoplasia of the anterior commissure [11]. Other defects included absence or hypoplasia of the pineal gland, cortical polymicrogyria, white matter changes in the corpus callosum and grey matter changes in the cerebellum [11-13]. Functional changes included hyposmia and abnormal inter-hemispheric auditory transfer [11,14].

The defining feature of all PAX proteins is the presence of a 128 amino acid DNA-binding paired domain that has been highly conserved over evolution [1]. In addition to the paired domain, PAX6 also contains a DNA-binding homeodomain and a proline, serine and threonine-rich (PST) domain at the C-terminus [1,6]. The PST domain, which encompasses the C-terminal 145 amino acids of PAX6, has been shown to act as a transcriptional activator [6]. The PAX6 protein directly regulates a wide range of target genes [1,2] including *Pax2 *[15], *Ngn2 *[16] and *glucagon *[17].

The *Pax6 *gene has a spatially and temporally complex expression pattern in the eye, brain, nasal structures, spinal cord and pancreas [1]. Although PAX6 is clearly involved in multiple developmental processes, common themes are now emerging concerning the role of PAX6 in neural tissues. Gradients of *Pax6 *expression are important for determining positional characteristics in the retina [18] and the neocortex [7]. PAX6 plays a role in development of specific axonal connections between the retina and the brain [18] and within the forebrain [8,19]. It is also involved in the differentiation of neural cell types from multipotent precursors in the retina [16] and the cerebral cortex [9] through activation of bHLH genes such as *Ngn2 *and *Mash1*. These studies provide a clear link between PAX6 function in the retina and the brain, and are of particular relevance to the neurological phenotypes of individuals with *PAX6 *mutations.

It is becoming apparent that transcription factors do not act in isolation but are dependent on interactions with other proteins to carry out their function [20,21]. These interactions introduce more specificity into the regulatory function of a given transcription factor. To date only three PAX6 protein-protein interactions have been described: with SOX2 on the lens-specific enhancer element of the δ-*crystallin *gene [22]; with MDIA, which modulates PAX6 activity in early neuronal development [23], and with MAF proteins on the *glucagon *promoter, which causes increased expression of this pancreatic hormone gene [17].

Here we report the preliminary results of the first systematic screen for proteins that interact with PAX6. We used sequence alignment algorithms and secondary structure prediction programs to define a new domain of 32 amino acids at the C-terminal end of the PAX6 protein. We then screened a brain library with this peptide using the yeast two-hybrid technique and identified three novel interacting proteins, HOMER3, DNCL1 and TRIM11. The interaction between PAX6 and these proteins was disrupted by naturally occurring C-terminal PAX6 mutations.

## Results

### A highly conserved C-terminal PAX6 peptide

**Figure 1:**
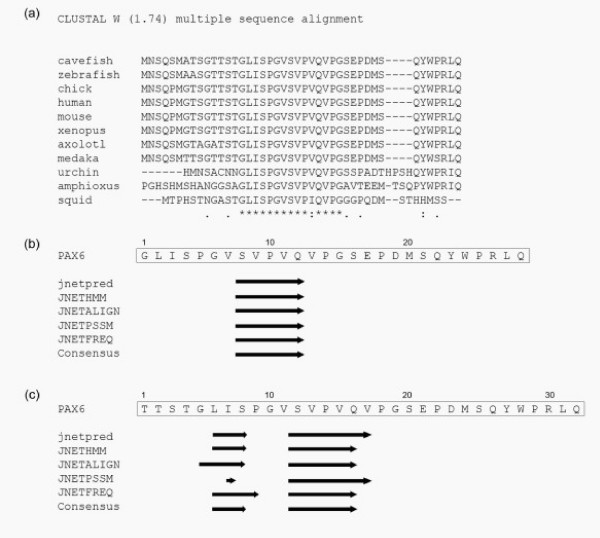
Characterisation of the PAX6 C-terminal peptide. (a) CLUSTAL alignment of the terminal 42 amino acids of PAX6 from diverse species. (*) indicates invariant residues, (:) indicates highly similar substitutions and (.) indicates moderately similar substitutions. (b) Secondary structure analysis of the highly conserved terminal 28 amino acids predicts a single beta sheet (arrow) in the SVPVQ peptide. (c) When 4 residues are added in the N-terminal direction, 2 beta sheets are now predicted.

We and others [31-33] noted that there is significant sequence conservation at the C-terminal end of the PAX6 protein. BLAST analysis of the amino acid sequence of the PAX6 PST domain (aa 278–422) revealed a highly conserved motif within the last 28 amino acids (beginning at the 'GLISP' motif, Figure 1a). Strong conservation was seen in distantly related species such as axolotl (*Ambystoma mexicanum*) and sea urchin (*Paracentrotus lividus*) (Figure 1a).

We subjected the whole PAX6 PST domain to secondary structure analysis using JPRED, a program that uses a number of different protein structure prediction algorithms to generate a consensus secondary structure (Figure 1) [25,26]. The PST domain was largely devoid of predicted secondary structure except for the C-terminal region, which contained two predicted beta sheets within the highly conserved domain, one in the 'GLISP' motif and one in the 'SPVPQ' motif (identical to the pattern shown in Figure 1c). Initially we defined the C-terminal domain as running from the 'GLISP' motif up to the stop codon, since this region was most highly conserved and contained strongly predicted secondary structure elements. However when we performed secondary structure prediction analysis on this 28 amino acid peptide, the first beta sheet was lost (Figure 1b). Addition of another 4 amino acids (TTST) immediately before 'GLISP' caused recovery of the first beta sheet (Figure 1c). Although these 4 residues are not highly conserved (Figure 1a), they appear to be important for seeding the first beta sheet and therefore for secondary structure. Thus we define the C-terminal peptide as being the final 32 amino acids of PAX6, running from threonine 391 to the stop codon (top line of Figure 1c).

### Yeast two-hybrid screening

We hypothesised that the C-terminal peptide might be involved in protein-protein interactions, and we tested this by screening a cDNA library using the yeast two-hybrid system with a construct (PAX6CTP) in which the 32 amino acid C-terminal peptide was fused to the yeast GAL4 DNA binding domain. We chose to screen a mouse brain cDNA library as no human libraries were available. Given the fact that the amino acid sequence of the PAX6 protein is identical in man and mouse, we reasoned that a mouse brain library would yield relevant interactors. For comparison we also carried out the screen using a construct containing the whole PST domain (PAX6PST).

The C-terminal peptide screen gave 15 colonies that were positive with all three reporters and the PST domain screen gave 62 colonies. The interacting plasmids were isolated and the cDNA inserts sequenced. Three cDNAs were identified 3 or more times, *Homer3 *(NM_011984), *Dncl1 *(Dynein cytoplasmic light chain 1, NM_019682, also known as *Pin *or *Dlc8*) and *Trim11 *(Tripartite motif protein family member 11, NM_053168). *Homer3 *(6 clones) and *Dncl1 *(2 clones) were identified in the C-terminal peptide screen. *Homer3 *(7 clones), *Dncl1 *(2 clones) and *Trim11 *(6 clones) were identified in the PST domain screen. All cDNA inserts were in-frame with the coding region of the pPC86 GAL4 activation domain. None of the cDNAs was present in a list of known false positives [34].

HOMER3 is a member of the HOMER family of neuronal post-synaptic density (PSD) proteins [35]. DNCL1 is a subunit of two motor protein complexes, dynein and myosin-Va, both of which are involved in intracellular trafficking of proteins and organelles in neurons [36,37]. TRIM11 is a member of the tripartite motif protein family and contains a RING finger, a B-box zinc finger, a coiled coil domain and a B30.2 domain [38]. The possible significance of the interactions between these proteins and PAX6 is discussed below.

### Semi-quantitative PCR

**Figure 2:**
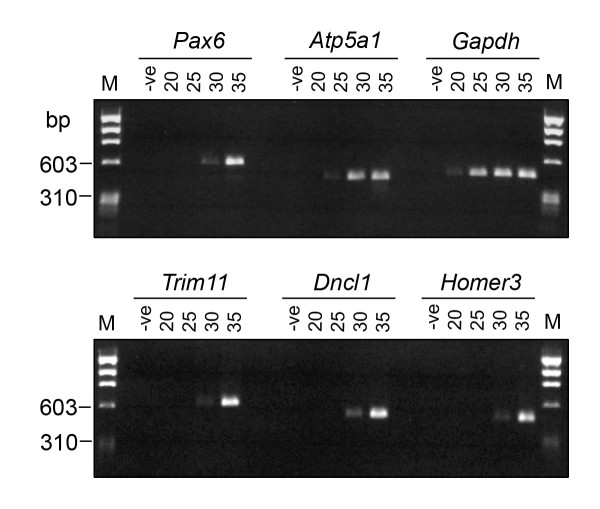
Semi-quantitative PCR analysis of *Homer3, Dncl1, Trim11 *and *Pax6 *in the mouse brain cDNA library. Library cDNA was amplified with primers specific for *Pax6 *(600 bp), *Homer3 *(485 bp band), *Dncl1 *(485 bp) and *Trim11 *(609 bp) for 20, 25, 30 or 35 cycles. *Atp5a1 *(415 bp) and *Gapdh *(450 bp), which are highly expressed in the brain, are included for comparison. M indicates the Φ×174 *Hae*III DNA size marker; the positions of the 603 bp and 310 bp marker bands are indicated.

To check that the *Homer3*, *Dncl1 *and *Trim11 *clones were not identified multiple times solely because they are highly abundant in the library, we performed a semi-quantitative PCR assay. We compared the relative abundance of *Homer3*, *Dncl1*, *Trim11 *and *Pax6 *with *Gapdh *and *Atp5a1*. *Gapdh *and *Atp5a1 *both show strong constitutive expression in a variety of tissues including the brain [29,30]. *Homer3*, *Dncl1*, *Trim11 *and *Pax6 *were only amplified strongly after 35 cycles of PCR (Figure 2) and were therefore present at relatively low levels compared to *Gapdh *(amplified strongly after 25 cycles) and *Atp5a1 *(amplified strongly after 30 cycles; Figure 2).

We concluded that *Homer3*, *Dncl1 *and *Trim11 *clones were not highly abundant in the library. This is consistent with the idea that they were pulled out because the encoded proteins interact specifically with the C-terminal peptide or PST domain of PAX6.

### Yeast two-hybrid pairwise interactions

By library screening we identified two proteins (HOMER3 and DNCL1) that interact with the C-terminal peptide and three proteins (HOMER3, DNCL1 and TRIM11) that interact with the whole PST domain. This suggests that HOMER3 and DNCL1 interact specifically with the C-terminal peptide while TRIM11 interacts with a more N-terminal part of the PST domain. We conducted pairwise tests between specific constructs to confirm the interactions identified in the library screen and to further investigate the interaction between PAX6 and HOMER3, DNCL1 and TRIM11. The *Dncl1 *and *Trim11 *clones that were pulled out of the library were full-length, but the *Homer3 *cDNAs lacked the N-terminal 70 amino acids. The missing coding region was inserted into the truncated *Homer3 *cDNA to give a full-length expression construct (see Methods). Pairwise interactions were carried out with both the full-length and truncated *Homer3 *clones.

**Figure 3:**
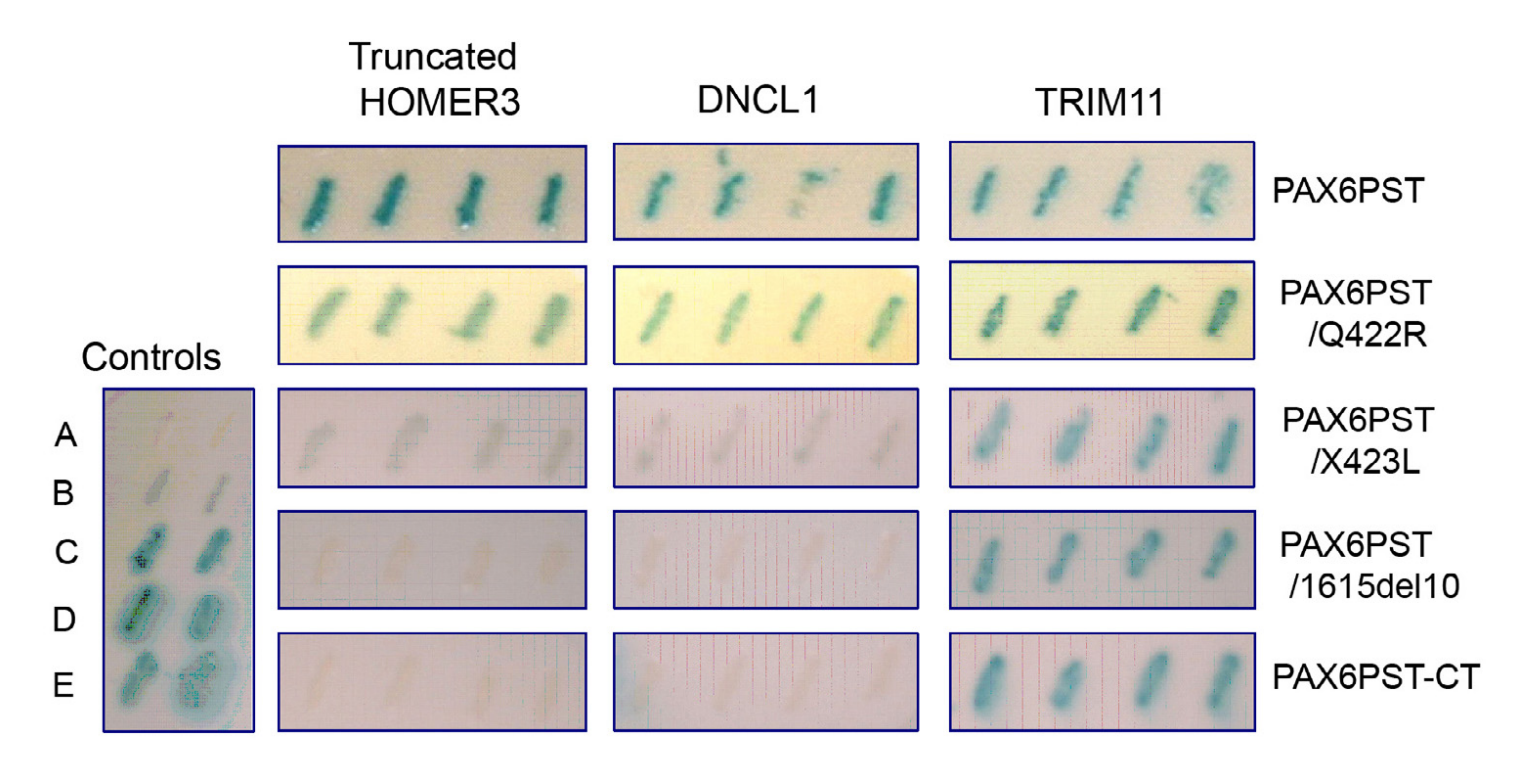
*LacZ *reporter gene activation in pairwise tests. pPC86 constructs are shown across the top, and pDBLeu constructs are shown down the right hand side. PAX6PST, PAX6 PST domain; PAX6PST/Q422R, PAX6 PST domain with the Q422R mutation; PAX6PST/X423L, PAX6 PST domain with the X423L mutation, PAX6PST/1615del10, PAX6 PST domain with the 1615 del10 mutation; PAX6PST-CT, PAX6 PST domain minus the C-terminal peptide. 'Truncated HOMER3' is HOMER3 lacking the N-terminal 70 amino acids. Five control strains are shown for comparison (left). These range from non-interactor (A) to strong interactor (E).

We confirmed that the whole PAX6 PST domain interacts with HOMER3 (full-length and truncated constructs), TRIM11 and DNCL1, as all three reporter genes were strongly activated in pairwise tests (Figure 3; Table 1). In contrast the interaction between the C-terminal peptide and HOMER3 or DNCL1 could not be confirmed with pairwise tests (Table 1). Occasionally, partial suppression of growth on plates containing 5-fluoro-orotic acid was observed, indicating low-level activation of the *URA3 *reporter; however *HIS3 *and *LacZ *activation were not observed. The reasons for this are not clear, although it may be that the pairwise tests were of sub-optimal sensitivity compared to the library screen. However we were able to confirm that the C-terminal peptide is important for the interaction with HOMER3 and DNCL1 because interaction with the PAX6PST-CT construct, which lacks the final 32 amino acids, was completely abolished (Figure 3, Table 1). TRIM11 interacted equally well with PAX6PST and PAX6PST-CT (Figure 3, Table 1). This is consistent with the library screens in which TRIM11 was isolated with the PST domain but not with the C-terminal peptide and supports the idea that the C-terminal peptide is not important for the interaction between PAX6 and TRIM11.

**Table 1:** Pairwise interaction tests between normal and mutant PAX6 constructs and HOMER3, DNCL1 and TRIM11. +++: strong interaction; ++: moderate interaction; +: weak interaction; (+) borderline interaction with one or two reporters activated at very low levels; 0: no interaction. PAX6PST, PAX6 PST domain; PAX6CTP, PAX6 C-terminal peptide; PAX6PST-CT, PAX6 PST domain minus the C-terminal peptide; PAX6PST/Q422R, PAX6 PST domain with the Q422R mutation; PAX6PST/X423L, PAX6 PST domain with the X423L mutation, PAX6PST/1615del, PAX6 PST domain with the 1615 del10 mutation. HOMER3-FL, HOMER3 full-length clone; HOMER3-Tr, truncated HOMER3 clone lacking the N-terminal 70 amino acids.

pDBLeu constructs	pPC86 constructs			
	HOMER3-FL	HOMER3-Tr	DNCL1	TRIM11
PAX6PST	+++	++	++	++
PAX6CTP	0	0	0	0
PAX6PST-CT	(+)	0	0	++
PAX6PST/Q422R	++	+	+	++
PAX6PST/X423L	+	0	(+)	++
PAX6PST/1615del10	(+)	0	0	++

Having confirmed that HOMER3, DNCL1 and TRIM11 interact with the PAX6 PST domain, we next investigated how the interactions were affected by three C-terminal PAX6 mutations that have been previously described in patients with ocular anomalies. The first mutation is a single nucleotide substitution 1627A>G that causes a glutamine to arginine amino acid substitution in the last codon of PAX6. This missense mutation (Q422R) has been reported in two patients, one affected by anterior segment dysgenesis with uveal ectropion and one with typical aniridia and foveal hypoplasia [27,33].

The second mutation (1615del10) was found in an aniridia family [28]. This frame-shifting deletion occurs just before the *PAX6 *stop codon and is predicted to cause translational read-through into the 3' untranslated region, generating a protein in which the last 5 amino acids of the C-terminal peptide are replaced by a 103 amino acid-extension. Affected individuals in this family showed unusual neurobehavioural traits including impaired social cognition and poor verbal inhibition [28]. MRI analysis revealed grey matter abnormalities in the frontal lobe, temporal lobe and cerebellum, and white matter deficits in the corpus callosum [13].

The third mutation 1629insT (X423L) has been reported in several aniridia patients [11,12,27,33]. Insertion of a single T nucleotide at position 1629 changes the stop codon (TAA) to a leucine codon (TTA) and generates a full length PAX6 protein with a C-terminal extension that extends for a further 35 amino acids into the 3' untranslated region. MRI analysis of six patients with this mutation revealed variable brain defects including absence or hypoplasia of the anterior commissure, pineal gland and olfactory bulbs [12]. Two patients had temporal polymicrogyria, one in association with epilepsy [12].

The three mutations were introduced into the PAX6PST construct, and pairwise tests were carried out to investigate the interaction of each mutant protein with HOMER3, DNCL1 and TRIM11. All three mutations had a clear effect on the interactions. The most subtle mutation (Q422R) caused a reduction in the interaction with HOMER3 and DNCL1 (Figure 3, Table 1). The C-terminal extension mutations X423L and1615del10 mutations both dramatically reduced or abolished the interaction with HOMER3 and DNCL1 (Figure 3, Table 1).

None of the three mutations affected the interaction with TRIM11 which again is consistent with the hypothesis that TRIM11 interacts with a more N-terminal part of the PST domain.

## Discussion

On the basis of secondary structure predictions and amino acid sequence conservation, we defined a novel PAX6 protein domain, which we have called the C-terminal peptide. We performed yeast two-hybrid library screens with the C-terminal peptide and the whole PST domain and we identified three novel interacting proteins, HOMER3, DNCL1 and TRIM11. In library screens, HOMER3 and DNCL1 interacted with the C-terminal peptide and the PST domain while TRIM11 interacted only with the PST domain, suggesting that HOMER3 and DNCL1 specifically interact with the C-terminal peptide while TRIM11 interacts with a more N-terminal part of the PST domain. The interactions between the PST domain and HOMER3, DNCL1 and TRIM11 were confirmed in pairwise tests. We were not able to confirm the interaction between HOMER3 or DNCL1 with the C-terminal peptide construct in pairwise tests, but we showed that the C-terminal peptide was important for PAX6/HOMER3 or PAX6/DNCL1 interaction because HOMER3 and DNCL1 did not interact with a PST domain construct lacking the C-terminal peptide.

HOMER3 is found in the PSD of neurons and directly binds to type I metabotropic glutamate receptors, which act via phospholipase C to stimulate IP3-mediated release of Ca^2+ ^from intracellular vesicles [35,39]. HOMER3 is a member of the HOMER family of proteins that are constitutively expressed in the brain and play a role in post-synaptic signalling and receptor trafficking by forming multivalent links with various receptors and PSD scaffolding proteins [35,39-41]. HOMER proteins have also been implicated in axon guidance during brain development [42].

DNCL1 is a subunit of two intracellular transport protein complexes, dynein and myosin Va [36]. Dynein and myosin Va are involved in the microtubule-based and actin-based movement respectively of proteins, organelles and vesicles in neurons [36,37]. Myosin Va is enriched in the PSD [43], and DNCL1 binds to a variety of PSD proteins including guanylate kinase domain-associated protein [44] and neuronal nitric oxide synthase [45].

TRIM11 is a member of the mouse tripartite motif protein family (also known as the RBCC family), and contains the three characteristic structural motifs of this protein family, a RING finger, a B-box zinc finger, and a coiled coil domain, as well as a B30.2 domain that is found in many but not all TRIM proteins [38]. TRIM11 interacts with Humanin, a protein that suppresses the neurotoxicity associated with Alzheimer's disease [46]. TRIM11 lowers Humanin levels by a mechanism that appears to involve ubiqutin-mediated proteasomal degradation [46].

At present our data must be considered preliminary because the interactions have not been confirmed by any other approach. However it is interesting to speculate that the interaction of PAX6 with HOMER3 and DNCL1 may be the basis of a mechanism by which synaptic signalling causes changes in gene expression. We propose that PAX6 is sequestered in the PSD by binding to HOMER3. Since receptor activation causes dissociation of HOMER proteins [35], PAX6 may be released as a result of synaptic activity, allowing it to interact with DNCL1. The PAX6/DNCL1 complex could then participate in myosin Va-mediated transport along the PSD-associated actin cytoskeleton followed by dynein-mediated transport along the microtubule network, eventually reaching the nucleus [36]. Since TRIM11 is implicated in protein degradation [46], it may play a role in PAX6 protein turnover.

Precedents for association of transcription factors with the post-synaptic density include STAT3 and CREB, which act as messengers between the synapse and the nucleus [47,48]. Although the full-length PAX6 protein is predominantly nuclear, there is good evidence in mouse, quail and nematode for an isoform that lacks the paired domain, is both nuclear and cytoplasmic, and binds DNA through the homeodomain alone [49-51]. In mice the paired-less isoform is relatively abundant in brain [49]; however its subcellular localisation in neurones, and the possibility of an association with the PSD, remains to be investigated.

Regarding the subcellular localisation of the putative interacting proteins, HOMER3 is predominantly found at the interface between the PSD and the cytoplasm [39], DNCL1 is chiefly cytoplasmic, although nuclear localisation has been reported [51], and TRIM11 is both nuclear and cytoplasmic [38]. Therefore cytoplasmic PAX6 could potentially interact with all three proteins, while nuclear PAX6 could interact with TRIM11 and DNCL1. At the tissue level, HOMER3 expression has been detected in thymus and lung but it has mainly been studied in brain where it is found in the forebrain, hippocampus and cerebellum [39]. DNCL1 and TRIM11 both have wide expression domains that include the brain [44,38]. Thus the expression of all three interactors overlaps with PAX6 at the tissue level [2,7,8,10]. We detected co-expression of *PAX6*, *HOMER3*, *DNCL1 *and *TRIM11 *by RT-PCR in human adult brain RNA (IH, L Harrison and A Brown, data not shown).

We demonstrated that the interaction between the PST domain and HOMER3 or DNCL1 was impaired by three naturally occurring mutations that are located in the PAX6 C-terminal peptide. The Q422R mutation, which involves a glutamine to arginine substitution at the last amino acid position of PAX6, caused a reduction in the interaction with HOMER3 and DNCL1. The X423L and 1615del10 mutations severely reduced or completely abolished the interaction with HOMER3 and DNCL1. The predicted effect of the X423L and 1615del10 mutations is to cause translation into the 3' untranslated region, thus generating proteins with abnormal extensions that might be expected to disrupt the conformation of the C-terminal end of the protein. The 1615del10 mutation also removes the last 5 amino acids of the C-terminal peptide [28].

None of the mutations affected the interaction with TRIM11, suggesting that they do not alter the conformation of the more N-terminal part of the PST domain. All our data are consistent with the hypothesis that TRIM11 does not interact with the C-terminal peptide, but interacts with the PST domain between the homeodomain and the C-terminal peptide.

Most aniridia patients are heterozygous for mutations that introduce a premature termination codon into the PAX6 open reading frame [2,52]. These alleles would be expected to encode truncated proteins or no protein at all if the mutant RNA is degraded by nonsense-mediated decay [52]. We propose that the brain anomalies that have been observed in aniridia patients may be partly explained by impaired interaction between PAX6 and HOMER3, DNCL1 and TRIM11. The neurobehavioural phenotype associated with 1615del10 and the polymicrogyria associated with X423L may result from a specific effect of these unusual C-terminal extension mutations. There is evidence that signalling and transport mechanisms that were initially characterized in the brain may also be conserved in the retina, suggesting that impaired PAX6 protein-protein interactions may also have implications for the retinal defects observed in individuals with *PAX6 *mutations [53,54].

## Conclusion

We have presented preliminary evidence that the neurodevelopmental transcriptional regulator PAX6 interacts with HOMER3, DNCL1 and TRIM11. We suggest that the interaction of PAX6 with HOMER3 and DNCL1 is a mechanism by which synaptic signalling could lead to regulated changes in gene expression in neurons. We also propose that some of the neural anomalies in patients with *PAX6 *mutations may be explained by impaired protein-protein interactions.

## Methods

### Bioinformatics techniques

Sequence database searches were carried out using the BLAST program available through the Bioinformatics Applications at the Rosalind Franklin Centre for Genomics Research [24]. Protein sequences that were highly homologous to the C-terminus of human PAX6 were aligned using CLUSTAL [24]. Secondary structure prediction was performed using the JPRED consensus method [25,26].

### Yeast two-hybrid constructs

All cDNA and amino acid numbering is based on the human PAX6 cDNA and protein reference sequences available from the Human PAX6 Allelic Variant Database web site [27]. Standard PCR and subcloning techniques were used to make three PAX6 cDNA constructs in the pDBLeu expression vector (ProQuest Two-Hybrid System, Invitrogen), which generates a protein fused to the yeast GAL4 DNA binding domain. PAX6PST contains the whole PST domain (amino acids 278–422 of the full-length PAX6 protein). Primers were ST001 (forward) 5'-AAA AGT TCG ACT GCC AGC AAC ACA CCT AGT C-3' and ST005 (R) 5'-TTT TGC GGC TTT TTA CTG TAA TCT TGG CCA GTA TTG-3'. PAX6CTP contains the newly defined C-terminal peptide alone (391–422). Primers were ST004 (F) 5'-AAA AGT CGA CTA CCA CTT CAA CAG GAC TCA TT-3' and ST005 (R). PAX6PST-CT contains the PST domain minus the C-terminal peptide (278–390). This was made by cutting PAX6PST with *Nde*I and *Not*I to drop out the C-terminal peptide, and inserting a synthetic linker between the two restriction sites. All fragments generated by PCR or with linkers were sequenced to check that no errors had been introduced.

A PAX6 PST domain construct containing the mutation 1627A>G (Q422R) was generated in the same way as the PAX6PST construct, but using the reverse PCR primer ST006 5'-TTT TGC GGC CGC TTT TTA CCG TAA TCT TGG CCA GTA TTG AG-3', which contains the mutant nucleotide substitution (underlined).

cDNA sequences containing the mutations 1615del10 [28] and 1629insT (X423L) [12] were generated by PCR from reverse transcribed RNA (a gift from Dr K Williamson and Prof V van Heyningen). Primers were ST015 (F) 5'-CCC ACA TAT GCA GAC ACA C-3' and ST031 (R) 5'-TTG CGG CCG CAT CCA TCC AGT CTA CAT TGT TC-3'. The PAX6PST construct was cut with *Nde*I and *Not*I to release the normal C-terminal peptide, and the mutant sequence was inserted.

### Yeast two-hybrid library screens

A mouse brain cDNA library (ProQuest, Invitrogen) was screened with the PAX6PST and PAX6CTP pDBLeu constructs. The library was constructed in the pPC86 vector, which produces proteins fused to the yeast GAL4 activation domain. The system uses three GAL4-activated reporter genes, *HIS3*, *URA3 *and *lacZ*, to identify positive interactions. Reporters are activated when the bait protein fused to the GAL4 DNA binding domain (pDBLeu) interacts with the prey protein fused to the GAL4 activation domain (pPC86), thus reconstituting GAL4 function. *HIS3 *activation allows growth on plates lacking histidine. Weak *URA3 *activation suppresses growth on plates containing 5-fluro-orotic acid while strong *URA3 *activation permits growth on plates lacking uracil. *LacZ *activation causes X-gal to turn blue in a beta-galactosidase assay. All assays were carried out in parallel with the five ProQuest control yeast strains A-E, which range from non-interactor (A) to strong interactor (E).

All procedures were carried out according to the supplier's protocols. Briefly, chemically competent MaV203 yeast cells were co-transformed with the cDNA library and the bait plasmid pDBLeu PAX6CTP or pDBLeu PAX6PST. Transformants (5 × 10^7^) were plated on medium lacking histidine to check for *HIS3 *activation. *HIS3 *positives were then assayed for all 3 reporters. The pPC86 prey plasmid was isolated from all *HIS3*/*URA3*/*LacZ *positives and the cDNA insert sequenced. BLAST searches were performed to identify the cDNA insert [24].

### Pairwise interactions

Specific interactions were tested by transforming competent MaV203 yeast cells with one bait construct (in pDBLeu) and one prey construct (in pPC86) and testing the resulting colonies for activation of the *HIS3*, *URA3 *and *LacZ *reporters as before.

To create a full-length *Homer3 *clone for pairwise tests, a cDNA clone (IMAGE clone 3602414, accession number BE569374) containing the missing N-terminal 70 amino acids was identified by a BLAST search of the EST nucleotide sequence database and obtained from the Rosalind Franklin Centre for Genomics Research. The missing fragment was amplified from the IMAGE clone by PCR and inserted into the pPC86-*Homer3 *plasmid to create a full-length expression construct.

### Semi-quantitative PCR

To check the relative representation of clones in the cDNA library, semi-quantitative PCR was performed on *Pax6*, *Homer3*, *Dncl1*, *Trim11 *and the constitutively expressed genes *Gapdh *and *Atp5a1 *[29,30]. Primers were designed to cross at least one intron, so that only correctly spliced clones were amplified. Primer sequences were: Pax6-F CAG CCA AAA TAG ATC TAC CTG; Pax6-R CGA TCA CAT GCT CTC TCC TT; Homer3-F CCC AGG TGG CTG TAG AGC; Homer3-R CTC TAC ACA GTG CAA AGC TCA G; Trim11-F GTG CAG GAT GTG AAG CTG; Trim11-R GCC TGC AGA TAG TCA TAG GG; Dncl1-F CAA AAA TGC AGA CAT GTC G; Dncl1-R CTA AGG GAG AAA AAA ATG GGG; Gapdh-F: CAT CAC CAT CTT CCA GGA GC; Gapdh-R: ATG ACC TTG CCC ACA GCC TT; Atp5a1-F: CAC ACG TGA GAT GTC CTC CA; Atp5a1-R: CAC AGA GAT TCG GGG ATA A. 10 ng library cDNA were amplified in a reaction containing 1xAmpliTaq polymerase buffer (Perkin Elmer), 1.5 mM MgCl_2_, 200μM each primer and 2.5 units of AmpliTaq polymerase (Perkin Elmer). PCR conditions were (95°C for 30 sec) × 1, (94°C for 30 sec, 55°C for 30 sec, 72°C for 30 sec) × 32 and (72°C for 2 min) × 1. Products were resolved on a 2.5% agarose gel with ΦX174/*Hae*III size markers (Promega).

## Abbreviations

PST domain, proline-, serine- and threonine-rich domain; PSD, post-synaptic density; PCR, polymerase chain reaction.

## Authors' contributions

STC carried out the bioinformatic analyses and all experimental work. IMH conceived, designed and supervised the study, and obtained funding. The manuscript was prepared jointly by STC and IMH, who have both read and approved the final version.
